# GLP-1 Analogs, SGLT-2, and DPP-4 Inhibitors: A Triad of Hope for Alzheimer’s Disease Therapy

**DOI:** 10.3390/biomedicines11113035

**Published:** 2023-11-12

**Authors:** Magdalena Złotek, Antonina Kurowska, Mariola Herbet, Iwona Piątkowska-Chmiel

**Affiliations:** Department of Toxicology, Faculty of Pharmacy, Medical University of Lublin, Jaczewskiego 8b Street, 20-090 Lublin, Poland; mzlotek00@gmail.com (M.Z.); akurowska123@gmail.com (A.K.); mariola.herbet@umlub.pl (M.H.)

**Keywords:** Alzheimer’s disease, antidiabetic drugs, neuroprotection, GLP-1 analogs, DPP-4 inhibitors, SGLT-2 inhibitors

## Abstract

Alzheimer’s is a prevalent, progressive neurodegenerative disease marked by cognitive decline and memory loss. The disease’s development involves various pathomechanisms, including amyloid-beta accumulation, neurofibrillary tangles, oxidative stress, inflammation, and mitochondrial dysfunction. Recent research suggests that antidiabetic drugs may enhance neuronal survival and cognitive function in diabetes. Given the well-documented correlation between diabetes and Alzheimer’s disease and the potential shared mechanisms, this review aimed to comprehensively assess the potential of new-generation anti-diabetic drugs, such as GLP-1 analogs, SGLT-2 inhibitors, and DPP-4 inhibitors, as promising therapeutic approaches for Alzheimer’s disease. This review aims to comprehensively assess the potential therapeutic applications of novel-generation antidiabetic drugs, including GLP-1 analogs, SGLT-2 inhibitors, and DPP-4 inhibitors, in the context of Alzheimer’s disease. In our considered opinion, antidiabetic drugs offer a promising avenue for groundbreaking developments and have the potential to revolutionize the landscape of Alzheimer’s disease treatment.

## 1. Introduction

Alzheimer’s disease presents an undeniable global challenge with profound societal, economic, and healthcare consequences. As the most prevalent form of dementia, it continues to escalate on a global scale. This neurological disease is characterized by a progressive degradation of brain function marked first by cognitive deficiencies, including loss of recent memory and language abilities and impairments in orienting, problem-solving, and abstract thinking [1]. In 2021, an estimated 50 million people worldwide grappled with Alzheimer’s, and this number is projected to triple by 2050, a surge intrinsically linked to the shifting demographics of an aging global population and increased longevity, which is associated with a heightened vulnerability to Alzheimer’s disease [2]. The development of Alzheimer’s is intricate, and influenced by a combination of genetic, environmental, and lifestyle factors. Notably, a family history of the disease is a significant risk factor, and specific genes, including the APOE gene, are associated with an increased risk of the condition [3]. Nevertheless, genetics alone do not solely determine one’s Alzheimer’s risk.

This disease’s complexity arises from the interplay of multiple pathomechanisms, including the accumulation of amyloid-beta, the formation of neurofibrillary tangles with hyperphosphorylated tau protein, oxidative stress, inflammation, mitochondrial disorders, and others. Patients with AD can manifest varying symptoms and disease severity due to individual differences in pathophysiology, making it challenging to define a universal treatment strategy [2,4].

Furthermore, the connection between Alzheimer’s disease and type 2 diabetes mellitus (T2DM) has gained recognition as an important risk factor for AD development [2]. While these are distinct diseases, research suggests shared risk factors and pathophysiological mechanisms, such as impaired insulin signaling, oxidative stress, inflammation, and the accumulation of amyloid-beta plaques [2]. Insulin resistance, a hallmark of T2DM, may have a role in the development of AD. The brain, like other organs, depends on insulin for various functions, including glucose metabolism and the regulation of neurotransmitters. Disruptions in insulin signaling may contribute to the pathology of AD, potentially affecting the accumulation of abnormal protein deposits in the brain, such as beta-amyloid plaques and tau tangles [5]. Just like diabetes patients, Alzheimer’s patients are also susceptible to inflammation and oxidative stress. Chronic inflammation and oxidative stress can harm brain cells and contribute to the progression of the disease [6].

While existing pharmacological treatments lower the symptoms of Alzheimer’s disease and enhance patients’ quality of life, they do not delay or cure the disease. Furthermore, the complex nature of AD makes early diagnosis challenging, often resulting in late-stage interventions. Currently, the FDA has approved only five medications for Alzheimer’s treatment: tacrine, donepezil, rivastigmine, galantamine, and memantine [1,7]. The first four are acetylcholine esterase inhibitors (AchEIs), while the fifth is an N-methyl-D-aspartate receptor antagonist (NMDARA). Although these drugs cannot stop the progression of the disease, they provide temporary relief by improving cognitive function through the partial amelioration of cholinergic and glutamatergic neurotransmission, particularly in the case of memantine [1,7]. Nevertheless, these medications can cause serious side effects, including muscle problems in anesthetic patients, slow heart rate, fainting, increased stomach acid levels, and seizures [1].

Nevertheless, there is reason for optimism in achieving a deeper understanding of the pathomechanisms underlying Alzheimer’s disease and in the quest for more effective treatments. This optimism is grounded in the ongoing progress in research technologies and the field of molecular biology.

Recent studies indicate that antidiabetic drug therapies can enhance neuronal survival and improve cognitive functions in diabetes by improving BBB integrity, reducing inflammation markers, limiting oxidative stress [8,9,10], and increasing the levels of neurotrophins [11]. Although the specific mechanisms in the context of AD remain incompletely understood, the undeniable link between the metabolic and molecular pathomechanisms shared by diabetes and Alzheimer’s disease underscores the urgency of investigating the potential therapeutic applications of antidiabetic drugs in the context of Alzheimer’s disease.

Our primary focus will be on elucidating the metabolic and molecular links between diabetes and Alzheimer’s disease and assessing the potential role of antidiabetic drugs including GLP-1 analogs, SGLT2 inhibitors, and DPP4 inhibitors, in the context of Alzheimer’s disease. We will delve into the mechanisms of action, evaluate their safety and effectiveness, and identify the challenges and future research directions in the pursuit of more effective treatments for this neurodegenerative condition.

## 2. Metabolic and Molecular Links between Diabetes and Alzheimer’s Disease

**Scheme 1:**
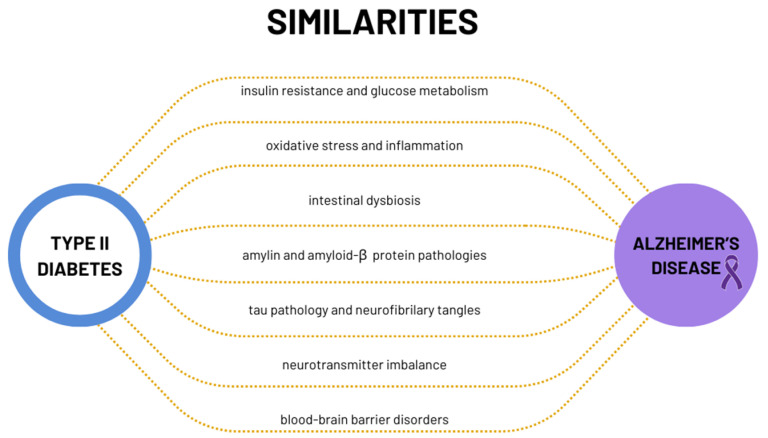
Metabolic and molecular similarities between type 2 diabetes and Alzheimer’s disease.

There has been a growing body of research suggesting that metabolic and molecular links exist between diabetes and Alzheimer’s disease (AD), highlighting potential interconnections between these seemingly distinct diseases (Scheme 1). While the precise nature of these links is complex and multifaceted, several hypotheses have arisen to illuminate the connection between diabetes and Alzheimer’s disease.

### 2.1. Insulin Resistance and Brain Glucose Metabolism

Insulin resistance is a complex phenomenon that occurs when cells in the body become less responsive to the effects of insulin, a hormone produced by the pancreas. Its primary function is to facilitate the uptake of glucose from the bloodstream into cells, where it can be used as an energy source or stored as glycogen in the liver and muscles [12]. Moreover, recent research has been uncovering a compelling connection between insulin resistance and brain health, highlighting its potential involvement in various neurological and cognitive disorders [13]. The brain, like other organs, requires a steady supply of glucose to function optimally. In fact, it is one of the most glucose-dependent organs in the body. Insulin resistance has been shown to disrupt the delicate balance of glucose utilization in the brain, potentially leading to adverse effects on cognitive function, memory, mood regulation, and even the risk of neurodegenerative diseases like Alzheimer’s disease [13]. Additionally, research has shown that insulin resistance can lead to a reduction in dendritic spine density and impair the formation of new synapses. This can result in altered synaptic plasticity, which is the brain’s ability to adapt and change in response to experiences [14]. Healthy dendritic spine formation and synapse development are crucial for maintaining optimal cognitive function, as they facilitate the transmission of information in the brain’s complex neural networks.

The compromised ability of brain cells to appropriately respond to insulin, observed in conditions like type 2 diabetes, could directly hamper their capacity to obtain energy and function optimally [13]. There is a growing body of evidence suggesting that AD may share metabolic and pathological similarities with T2DM. This has led to the concept of “brain-related type 2 diabetes” or “type 3 diabetes”, highlighting the potential role of insulin resistance in brain-related diseases. Studies have demonstrated reduced brain glucose utilization in AD patients [15]. These include disruptions in glucose transport, such as insulin resistance and anomalous glucose transporter activity [16], as well as alterations in blood flow dynamics [17]. Intracellularly, abnormalities involve cytosolic processes, notably glycolysis and the pentose phosphate pathway, alongside anomalies in mitochondria-dependent processes like the tricarboxylic acid (TCA) cycle and oxidative phosphorylation [18].

In addition, PET imaging studies have brought new insight into the relationship between amyloid plaque deposition and glucose metabolism. For carriers of autosomal dominant mutations predisposing to Alzheimer’s disease (AD), a fascinating sequence of events has been observed. The deposition of amyloid beta (Aβ), which is the hallmark of AD plaques, begins 15–25 years before the expected onset of disease symptoms. In contrast, glucose hypometabolism in specific areas of the brain appears about 5–10 years later, constituting the first Aβ biomarker. It is concluded that this hypometabolism may be the result of Aβ deposition in the pathogenetic mechanisms of Alzheimer’s disease [19,20].

Furthermore, insulin resistance can lead to dysregulation of insulin signaling pathways within the brain. When these signaling pathways are disrupted, they can influence cognitive processes and potentially contribute to the development of cognitive impairments [21].

Insulin resistance has also been associated with brain atrophy, particularly in areas of the brain responsible for cognitive functions [13]. The reduction in brain tissue volume contributes to cognitive decline and neurodegeneration. Reduced metabolism has been observed in the parietal, temporal, and frontal cortical brain areas important for cognitive function and implicated in AD development [22].

Insulin also contributes to blood–brain barrier (BBB) integrity. When insulin resistance occurs, the BBB may be compromised, potentially allowing harmful substances to enter the brain, and contributing to inflammation and neurodegeneration [23]. Insulin signaling affects amyloid precursor protein (APP) processing, favoring the non-amyloidogenic pathway in proper signaling, while disruptions shift it toward the amyloidogenic pathway, increasing Aβ production linked to cognitive decline [24,25,26,27]. Zhao et al.’s research supports insulin’s role in converting Aβ oligomers into less harmful monomers, protecting neurons from amyloid plaque toxicity [28].

Furthermore, the influence of insulin dysfunction on Alzheimer’s disease (AD) pathogenesis has been underscored by studies involving mice with streptozotocin-induced (STZ) type I diabetes. These studies have indicated a noteworthy increase in the accumulation of Aβ plaques within the brain, which in turn could initiate a cascade of inflammatory responses [29]. An additional layer of complexity emerges from the work of Devi et al., who have delved into the effects of insulin deficiency. Their findings illuminate the impact of insulin insufficiency on the activity of β-secretase—an enzyme intricately linked to the formation of amyloid plaques. Disruptions in insulin signaling, as reported by Devi et al., could potentially disturb the delicate balance of this enzyme’s activity, fostering an environment conducive to the excessive buildup of amyloid plaques [30]. This sheds light on yet another avenue through which insulin dysfunction might contribute to the pathological processes underpinning AD.

Furthermore, insulin resistance can lead to chronic inflammation, oxidative stress, and mitochondrial dysfunction in the brain. These processes can initiate cascades of cell death and inflammation, contributing to the pathology of neurodegenerative diseases like AD [14].

### 2.2. Oxidative Stress and Inflammation

Oxidative stress and inflammation are another bridge between two seemingly disparate conditions: diabetes and Alzheimer’s disease (AD). Oxidative stress, characterized by an imbalance between the production of harmful reactive oxygen species (ROS) and the body’s antioxidative defenses, is a common weave in the fabric of diabetes and AD. In diabetes, prolonged high levels of glucose not only reduce the activity of antioxidants but also lead to overproduction of reactive oxygen species (ROS). These free radicals can cause oxidative stress, which poses a threat to the proper functioning of tissues. The brain is particularly susceptible to oxidative damage due to its high oxygen consumption, high lipid content, and relative deficiency of antioxidant enzymes compared to other tissues [31]. Additionally, in diabetes, the brain experiences even more reduced activity of antioxidant enzymes such as superoxide dismutase and catalase, which translates into the level of oxidative stress in neurons [32,33]. In addition, glucose autoxidation and lipid peroxidation enhance this process [34]. The reduced concentration of antioxidants, including reduced glutathione, instigates mitochondrial oxidative harm, potentially culminating in apoptosis and/or necrosis of cells and inhibition of neurogenesis [35,36]. This collectively contributes to neurological complications in people with diabetes.

Similarly, the critical aspect that contributes to the pathogenesis of AD is the generation of reactive oxygen species (ROS), dysfunctional mitochondria, and accumulation of amyloid-β plaques [37]. Mitochondrial dysfunction in AD is linked to multiple factors, including genetic mutations, impaired mitochondrial dynamics, and disrupted electron transport chain activity. These factors can cause leaks in the electron transport chain, leading to the production of ROS as a byproduct. AD patients show elevated oxidative damage to brain macromolecules like proteins, sugars, lipids, and nucleic acids [38]. Elevated markers of protein oxidation, glycation, and glycoxidation is common in AD and MCI brain tissue [39]. Lipid peroxidation products, including 4-hydroxynonal, malondialdehyde (MDA), and acrolein, are elevated in multiple AD- and MCI-affected brain regions [40]. Oxidative damage to DNA and RNA is evident through higher levels of 8-hydroxydeoxyguanosine (8-OHdG) and 8-hydroxyguanosine (8-OHG), particularly in DNA (including mitochondrial DNA) [41].

It has been established that mitochondrial dysfunction is an early hallmark of AD, preceding the clinical symptoms [39]. Reduced cerebral oxygen metabolism, particularly in the frontal, parietal, and temporal cortex, correlates with the severity of dementia in AD [42,43].

Neuroinflammation serves as a common denominator in both diabetes and Alzheimer’s disease. These excess ROS play a crucial role in initiating chronic inflammation. The inflammatory response triggered by ROS in diabetes involves the activation of pro-inflammatory signaling pathways, such as nuclear factor-kappa B (NF-κB). NF-κB regulates the expression of numerous genes involved in inflammation, and its activation is closely linked to the development of insulin resistance—the hallmark of type 2 diabetes [44,45,46]. In addition, ROS-mediated inflammation can also lead to dysfunction of the endothelial cells lining blood vessels, contributing to vascular complications commonly seen in diabetes, such as atherosclerosis and impaired blood flow [47].

Similarly, in Alzheimer’s disease, oxidative stress amplifies the inflammatory response within the brain. ROS can activate microglia, which are the immune cells of the central nervous system, leading to the release of pro-inflammatory cytokines and other signaling molecules. This neuroinflammatory response contributes to synaptic dysfunction and neuronal damage, further impairing cognitive function [48]. Additionally, oxidative stress can directly impact the functioning of neurons, affecting their ability to communicate and form functional neural networks, which are vital for memory and cognitive processes [49,50,51].

### 2.3. Intestinal Dysbiosis

Dysbiosis, an imbalance in gut microbial composition, is observed in both Alzheimer’s disease (AD) [52] and diabetes [53,54,55]. The gut microbiota (GM), far from being confined to digestion, profoundly impacts overall human health including the functions of the central nervous system [56].

An increasing body of research has highlighted the role of the gut microbiota in modulating the oxidative/antioxidant equilibrium within the central nervous system (CNS) [57,58,59].

GM actively regulates nitric oxide (NO) and reactive oxygen species (ROS) production in the gut, which happens with the participation of bacteria like Lactobacilli, Bifidobacterium, and Streptococcus [60,61]. It is widely acknowledged that NO at nanomolar levels exerts neuroprotective effects, yet elevated NO concentrations may induce oxidative stress, intricately tied to axonal degeneration, neuroinflammation, and neurodegenerative disorders (NDs) [56]. In addition to this, certain pathogenic bacteria like Salmonella and Escherichia coli possess the capability to produce hydrogen sulfide (H_2_S) through the breakdown of sulfur-containing amino acids in the gut. Elevated H_2_S levels alter multiple host metabolic pathways, resulting in increased lactate, decreased oxygen consumption, diminished ATP production, and heightened proinflammatory compound levels. These changes have been linked to processes of neuroinflammation and neurodegeneration [62].

Notably, type 2 diabetes (T2DM) patients exhibit a dysbiotic profile, marked by a decline in beneficial butyrate-producing bacteria (Firmicutes, Roseburia, Faecalibacterium prausnitzii) and a rise in opportunistic bacterial strains [63]. Research shows that the dysbiosis of microflora described in diabetic patients promotes the increase in insulin resistance [64]. Interestingly, research suggests that obesity, which is common in T2DM patients, affects short-term and working memory through the influence of the gut microbiota on the metabolism of aromatic amino acids [65].

The intricate connection between Alzheimer’s disease and metabolic changes suggests the gut microbiota’s a significant role. Disruption of the gut barrier function, often due to an excess of pro-inflammatory bacteria and a decrease in anti-inflammatory counterparts, permits the entry of inflammatory molecules into the bloodstream. This fosters chronic inflammation and disrupts the blood–brain barrier, a hallmark of diabetes [66,67].

Dementia and amyloidosis patients exhibit elevated pro-inflammatory cytokines in their blood, associated with increased pro-inflammatory gut bacteria [67]. Certain types of gut microbiota can produce or metabolize neurotransmitters such as γ-aminobutyric acid (GABA) [68], which plays a key role in brain aging [69]. Further, studies suggest a connection between altered gut microbiota, systemic inflammation, and the accumulation of amyloid-beta in the brain [70].

### 2.4. Amylin and Amyloid-β (Aβ) Protein Pathologies

The interplay between amylin and amyloid-β (Aβ) protein pathologies is an intriguing area of study that sheds light on the complex relationship between neurodegenerative diseases like Alzheimer’s disease (AD) and metabolic disorders such as type 2 diabetes (T2D). Amylin, also known as islet amyloid polypeptide (IAPP), and Aβ are two distinct protein aggregates associated with different diseases but sharing similarities in terms of their aggregation properties and potential impact on neuronal health.

Amylin is a peptide hormone primarily produced by the pancreatic beta cells. It plays a role in regulating glucose metabolism and satiety. However, in conditions like T2D, amylin can misfold and aggregate, leading to the formation of amyloid deposits (islet amyloid peptide—IAPP) within the pancreas. These amyloid deposits contribute to pancreatic beta cell dysfunction and decreased insulin secretion, characteristic features of T2D. Such a correlation may lead to disruptions in the regulation of glucose metabolism, creating a favorable ground for the evolution of processes related to neurodegenerative diseases [71]. Interestingly, similar amyloid deposits composed of amylin have been identified in the brains of individuals with T2D and Alzheimer’s disease [72,73,74].

In AD, the accumulation of Aβ plaques is a hallmark feature, contributing to synaptic dysfunction and neurodegeneration. There is emerging evidence suggesting a link between amylin and Aβ pathologies. Studies have shown that amylin can cross the blood–brain barrier and interact with Aβ in the brain. Amylin might contribute to the formation of Aβ plaques and exacerbate AD-related pathology [75]. Further, research has indicated that amyloid-beta (Aβ) can engage in direct interactions with metals found in the brain, such as copper and iron. This interaction sets off a chain of events that culminates in the generation of reactive oxygen species (ROS) through mechanisms akin to the Fenton reaction. Additionally, Aβ-induced dysfunction within mitochondria, the cellular powerhouses, further intensifies the production of ROS. This creates a detrimental feedback loop, where elevated ROS levels contribute to an augmented aggregation of Aβ, and reciprocally, the accumulation of Aβ fosters more ROS production. This intricate interplay was highlighted by a study conducted by Southam and colleagues in 2016 [48]. Mitochondrial dysfunction and Aβ aggregation are closely linked to synaptic deficits observed in AD [76,77].

### 2.5. Tau Pathology and Neurofibrillary Tangles

From a pathological perspective, the brain of an individual with Alzheimer’s disease (AD) not only exhibits the accumulation of Aβ aggregates but also the presence of neurofibrillary tangles. Tau, a protein closely linked with microtubules, constitutes the primary element of neurofibrillary tangles (NFTs) [78,79]. Hyperphosphorylation of tau within the brains of Alzheimer’s disease patients has been associated with a deficiency in insulin signaling within the brains of individuals with type 2 diabetes (DM) [78].

Hyperphosphorylation of tau within the brains of Alzheimer’s disease (AD) patients has been associated with a deficiency in insulin signaling within the brains of individuals with type 2 diabetes (DM) [78]. Glycogen kinase-3β (GSK-3β), a key player in insulin signaling, is implicated in driving tau hyperphosphorylation. Glycogen kinase-3β (GSK-3β) may be a link between diabetes and Alzheimer’s [80,81,82]. Disrupted insulin signaling in diabetes affecting the GSK-3β pathway can lead to excessive tau phosphorylation, increasing the risk of Alzheimer’s. Ongoing research seeks to understand this link.

### 2.6. Neurotransmitter Imbalance

Emerging evidence suggests that changes in the delicate balance of neurotransmitters inside the brain can play a key role in the development and progression of diabetes and Alzheimer’s disease [83]. AD is now recognized as a type 3 diabetes due to neuroendocrine aspects [84].An increasing body of research suggests that, in addition to decreased insulin signaling, a variety of other variables may operate as mechanistic linkages between AD and T2DM [83]. Hypercholesterolemia, dyslipidemia, hypercystinemia, inflammation, poor insulin signaling, and impaired central nerve response to the adipose-tissue-derived hormone leptin are the main culprits. Increased cholesterol is crucial in amyloid precursor protein metabolism and accumulation [84]. 

Diabetes, in addition to impairing insulin signaling, has been linked to an increase in cerebrovascular inflammation and amyloid peptide (A) deposition. The formation of advanced glycation end products and increased oxidative stress are critical in the balance of neurotransmitters. Dysregulation of neurotransmitters like insulin and insulin-like growth factor-1 (IGF-1) could impact synaptic plasticity and cognitive function [85]. The disruption could further contribute to cognitive deficits in AD.

Furthermore, the cognitive impairment associated with Alzheimer’s disease is a result of lower levels of acetylcholine in the brain, which results from dysfunction of cholinergic neurons [86]. In vivo studies in older rats have shown that an increase in the concentration of IGF-1 in the hippocampus to the level found in young animals reverses memory and learning deficits. These studies may be important and give evidence that a decrease in IGF-1 levels may correlate with the pathogenesis of AD, which mainly affects the elderly [87]. Also, Carro et al. in a study in mice showed that the deletion of the IGF-1 gene causes a premature increase in the level of Aβ in the brain, characteristic of AD pathogenesis [88].

It is worth noting that metabolic changes in T2D also disturb the balance between glutamate and GABA, causing cognitive impairment [89]. Emerging evidence suggests that changes in the delicate balance of neurotransmitters inside the brain can play a key role in the development and progression of this debilitating ailment.

### 2.7. Blood–Brain Barrier Disorders

The blood–brain barrier (BBB) regulates the molecular exchange between blood flow and brain parenchyma, thereby regulating central nervous system (CNS) homeostasis [90,91]. As a result, BBB dysfunction is associated with several neurological conditions [90]. Diabetes often leads to vascular complications, impaired blood flow, disruption of the blood–brain barrier, and endothelial dysfunction. Similar vascular issues can also be observed in Alzheimer’s disease (AD), involving reduced endothelial transport, loss of tight junction integrity, basement membrane disorganization, pericyte degeneration, and astrocyte depolarization [90]. These conditions can promote neuronal damage and contribute to the development of AD. A neuropathological investigation found massive infarcts, lacunae, many microinfarcts, hemorrhaging, atherosclerosis, and arteriolosclerosis in 80% of Alzheimer’s disease patients [92]. BBB dysfunction may also allow the entry of toxic molecules and inflammatory factors into the brain, further contributing to AD pathology, as shown in animal models and patients with AD-related diseases [90,93]. Simultaneously, the breakdown of the BBB leads to plasma protein leakage, diminished brain glucose absorption, and neuroinflammation, as highlighted by Yamazaki and Kanekiyo in 2017 [94]. This compromised BBB integrity may result in increased cellular toxicity, rendering neurons more vulnerable to the ravages of toxins. Cerebral amyloid angiopathy (CAA) emerges as a significant vascular comorbidity within the context of Alzheimer’s disease (AD). This condition involves the accumulation of amyloid (A) in the walls of arteries, arterioles, and, to a lesser extent, capillaries, leptomeninges, and cortical regions, as elucidated by Dallaire-Théroux et al., 2017 [95]. While it is suggested that CAA results from a failure in A clearance from the brain, the underlying mechanisms remain enigmatic. CAA is prevalent in the majority (80%) of AD cases, but it can also manifest in 10–40% of the general population. Notably, carriers of the apolipoprotein E (APOE) ε4 allele, a well-established genetic risk factor for AD, tend to exhibit more severe CAA. Additionally, CAA stands as a pathogenic hallmark in hereditary dementia disorders when different variants of the accumulating peptide are identified, as indicated by Klohs in 2020 [96]. A better knowledge of how BBB failure contributes to or is caused by AD development could help develop diagnostic and treatment methods for this devastating illness.

## 3. From Blood Sugar Control to Brain Health: The Potential Role of Antidiabetic Drugs in Alzheimer’s Disease

### 3.1. GLP-1 Receptor Agonists

#### 3.1.1. Mechanism of Action GLP-1 Receptor Agonists

In the early 1980s, a pivotal discovery shook the realm of diabetes research. Scientists stumbled upon a remarkable revelation: Glucagon-like peptide 1 (GLP-1), a hormone produced in the gut, wields an uncanny ability to regulate blood glucose levels. This discovery sparked great interest among researchers for its potential use in diabetes therapy [97,98]. However, it took until the year 1992 for a momentous breakthrough to occur—the successful cloning and identification of the GLP-1 receptor [97]. This landmark achievement embarked the development of GLP-1 receptor agonists. Now, GLP-1 analogs represent an innovative alternative to traditional oral antidiabetic drugs, offering superior glycemic control. They achieved this by enhancing insulin secretion and inhibiting the release of glucagon, both crucial factors in glucose management [99,100]. GLP-1 receptor agonists have the added benefit of promoting weight loss. This effect is particularly beneficial for individuals with type 2 diabetes who are overweight or obese [101]. Clinical studies suggested that the use of GLP-1 receptor agonists is associated with a decreased risk of cardiovascular complications, which are a significant concern for individuals with diabetes [102]. The secret to the success of GLP-1 receptor agonists lies in their unique structural modifications. While these analogs show a striking resemblance to the natural GLP-1 hormone, they were intentionally engineered to resist degradation by dipeptidyl peptidase-4 (DPP-4), an enzyme that rapidly breaks down native GLP-1. These modifications translated to more potent and sustained effects on blood glucose regulation, making them highly effective weapons in the fight against diabetes [103,104,105] (Table 1).

#### 3.1.2. Physiological and Pharmacokinetic Attributes of GLP-1 Receptor Agonists in Central Nervous System—Mechanisms and Benefits

GLP-1 receptor agonists (GLP-1RAs), beyond their well-established role in regulating blood sugar levels, represent a promising area of research related to their effects on brain function, and they can demonstrate benefits in the context of Alzheimer’s disease and Parkinson’s disease [122,123,124,125]. GLP-1 analogs possess a minimal risk of causing hypoglycemia, which makes them potentially appropriate as safe therapies for non-hyperglycemic conditions like neurological disorders [122]. GLP-1 receptors are widely distributed in the body, expressed on pancreatic beta cells and neurons of the central and peripheral nervous system [126,127,128]. GLP-1 receptors are abundantly expressed in the periventricular organs (CVO) as well as in the nuclei of the brain, which play a key role in the regulation of energy balance, including the control of food intake and energy expenditure, as well as in glucose metabolism [129,130]. Studies show that stimulation of the GLP-1 receptor in the solitary nucleus leads to the inhibition of the glucose production process, with no effect on its uptake. This significant finding highlights the potential role of the central GLP-1 system in regulating both energy balance and glucose metabolism through the GLP-1 analogs [131,132].

This range provides a balance between the ability to cross biological membranes, including the blood–brain barrier (BBB), and avoiding excessive binding to plasma proteins. GLP-1RAs’ capacity to traverse the blood–brain barrier, combined with the meticulous attention to lipophilicity, firmly establishes them as leaders in the pursuit of effective therapies for central nervous system disorders. A study by Kastin and Akerstrom produced groundbreaking findings, indicating that Exendin-4, administered peripherally, is able to cross the blood–brain barrier relatively easily [133]. This effect facilitates its access to the brain, and Exendin-4’s lipophilicity plays a key role here. Liraglutide, exenatide, and lixisenatide have low-molecular-weight lipid-soluble molecules (less than 5kDa) that are expected to pass through the BBB, unlike native GLP-1, which is degraded by DPP-4 in approximately 5 min after being absorbed [106].

Studies suggest that semaglutide may bind to albumin and directly reach the brainstem, septal nucleus, and hypothalamus, but it does not have the ability to bind to endothelial cells, which makes it difficult to penetrate the BBB. However, it can penetrate the brain through areas lacking the BBB [106]. Dulaglutide has a much higher molecular weight than other GLP-1 analogs, which may contribute to the lack of direct evidence that dulaglutide crosses the BBB [106]. It is important to remember that challenges related to crossing the blood–brain barrier (BBB) persist under normal physiological conditions. This situation remains changed when considering instances where barrier integrity is compromised, with inflammation as a prime example. Some preclinical studies and small-scale clinical trials have suggested that GLP-1 receptor agonists may have neuroprotective properties [6,122,134,135,136]. These drugs appear to have a positive effect on neuronal function and survival, potentially reducing the risk or slowing the progression of neurodegenerative diseases. GLP-1 receptor analogs have been demonstrated to protect existing synapses and promote the formation of new ones, providing additional support for their potential as Alzheimer’s therapeutics.

Moreover, emerging evidence suggests that GLP-1 receptor agonists are not only effective in preventing the accumulation of amyloid beta, a protein commonly associated with Alzheimer’s disease, but also in mitigating the hyperphosphorylation and aggregation of another critical protein in the disease’s progression: tau. Furthermore, these compounds have shown the ability to enhance synaptic plasticity in the hippocampus, a region crucial for memory and cognitive function. The process of reduced Aβ production in the presence of GLP-1 analogs may be mediated by increased expression of the insulin-degrading enzyme (IDE) in the brain, which promotes Aβ breakdown [137,138], as well as by reduced activity of JNK (c-Jun N-terminal kinase) and BACE1, enzymes involved in the production of Aβ [138,139,140]. In addition, studies show that GLP-1R agonists normalize the activity of the enzyme N-acetylglucosaminyltransferase III (GnT-III) and the levels of N-acetylglucosamine (GlcNAc) in the brain, which inhibits processes related to the production of Aβ [141].

In addition to their role in protein aggregation and synaptic health, GLP-1 receptor mimetics have displayed the capacity to improve insulin sensitivity, a critical factor in the context of Alzheimer’s disease. Insulin resistance in the brain has been linked to cognitive decline and neurodegeneration. GLP-1 receptor agonists have been shown to enhance insulin sensitivity in both peripheral tissues and the central nervous system, potentially mitigating the detrimental effects of insulin resistance on brain function. Moreover, in vivo studies have unveiled anti-inflammatory effects associated with GLP-1 receptor agonists, further underscoring their potential in addressing the chronic neuroinflammation often observed in Alzheimer’s patients. By modulating neuroinflammatory responses, GLP-1 receptor mimetics hold the promise of reducing the harmful immune activation that contributes to neuronal damage and cognitive decline in Alzheimer’s disease [124,137,142].

#### 3.1.3. Molecular Mechanisms Underlying the Neuroprotective Effects of GLP-1 Agonists’ Improvement of Insulin Sensitivity

There is a mounting body of evidence pointing to a direct association between Alzheimer’s disease (AD) and insulin resistance within brain cells. This phenomenon hinders the efficient utilization of glucose. It disrupts energy metabolism within neurons, thereby compromising their ability to generate adenosine triphosphate (ATP), a crucial molecule for cell survival and proper function [12,14]. Moreover, insulin resistance is believed to exacerbate oxidative stress and trigger neuroinflammatory processes within brain cells [14]. As insulin resistance progresses, it can lead to cognitive impairments, including memory deficits and difficulties with spatial awareness and problem-solving—hallmarks of AD. Interestingly, GLP-1 analogs, initially developed to manage diabetes, have piqued the interest of researchers due to their potential to enhance insulin sensitivity within the central nervous system (CNS). GLP-1 agonists act by binding to GLP-1 receptors on pancreatic β-cells and stimulate the release of insulin from pancreatic β-cells in a glucose-dependent manner. In addition to this, GLP-1 agonists also enhance insulin biosynthesis at the translational level, through the mechanism of activation of cAMP/PKA-dependent and independent signaling pathways, as well as increases in intracellular Ca^2+^ levels, which help to maintain the insulin stores in β-cells and their ability to secrete insulin. PKA promotes the release of insulin by increasing the sensitivity of beta cells to glucose [98,143]. Furthermore, GLP-1 enhances the ability of glucose-resistant β-cells to sense and respond to glucose by conferring glucose sensitivity. This results in an improvement in the capacity of β-cells to effectively respond to changes in glucose levels [98,105,143]. Batista et al., in their research on in vivo and in vitro models, demonstrated that liraglutide prevents the loss of brain insulin receptors and synapses by activating the PKA signaling pathway [144]. The study results have led to the conclusion that liraglutide has a protective effect on insulin receptors (IR), suggesting that combination therapy using liraglutide to prevent the decline of IR along with insulin may be an attractive therapeutic approach in neurodegenerative diseases.

##### Anti-Inflammatory Effects

In neurodegenerative conditions like Alzheimer’s disease (AD), chronic inflammation plays a pivotal role in the disruption of the blood–brain barrier, primarily driven by signaling molecules such as TNFα and IL-1β. This breach in the barrier allows immune cells to infiltrate the central nervous system (CNS), triggering a cascade of detrimental effects. This includes mitochondrial and axonal damage, dysfunction at synapses, and the activation of key cellular players like microglia, astrocytes, and neurons, ultimately culminating in cellular dysfunction and, in some cases, cell death [55,108]. Numerous studies have consistently shown that GLP-1R agonists like liraglutide, exenatide, and lixisenatide possess the capacity to mitigate neuroinflammation in models of Alzheimer’s disease (AD), leading to an improvement in cognitive function. These findings collectively underscore the substantial potential of GLP-1R agonists in attenuating neuroinflammation-associated cognitive deficits and ultimately enhancing cognitive performance [145,146,147,148]. In AD, the activated microglial astrocytes play significant roles in the inflammation of AD pathogenesis. Some findings show that these drugs can function to modulate inflammation relevant to microglia in AD brains [147]. Parthasarathy and colleagues discovered that liraglutide, a GLP-1R agonist, significantly diminishes the presence of activated microglia in the cortex and dentate gyrus region of the hippocampus, as well as activated astrocytes in the cortex. Additionally, the levels of pro-inflammatory cytokines such as IL-6, IL12p70, and IL-1β were markedly reduced in the brains of mice treated with liraglutide [148]. Another study reported that prophylactic administration of liraglutide effectively curtails chronic inflammation characterized by activated microglia in the cortex and concurrently prevents memory impairment in APP/PS1 mice [149].

Exendin-4 and liraglutide have undergone thorough examination in Alzheimer’s disease (AD) models, demonstrating their efficacy in reducing inflammation, enhancing mitochondrial function, providing neuroprotection, and ameliorating memory and behavioral deficits [138]. NLY01, a GLP-1R agonist developed based on exendin-4, has exhibited the remarkable ability to selectively inhibit microglia activation triggered by β-amyloid (Aβ) via GLP-1R activation in AD models [138]. Remarkably, Paladugu et al. found that liraglutide treatment proved more effective in reducing neuroinflammation and restoring disrupted insulin signaling in prodromal streptozotocin (STZ)/sporadic AD mice compared to prodromal 5×FAD (five AD-linked mutations) mice. These results suggest that liraglutide holds potential as a therapeutic agent with anti-inflammatory and anti-amyloid properties, potentially delaying symptom onset during the prodromal stages of AD, especially in the sporadic form of the disease [137]. Furthermore, GLP-1RAs have exhibited neuroprotective effects in various animal models of acute CNS injuries, including stroke or traumatic brain injury [136]. Excitingly, phase III clinical trials are currently underway with semaglutide in early-stage Alzheimer’s disease [150]. Semaglutide is believed to exert its neuroprotective effect through a variety of mechanisms, including the modulation of nerve cells and other cell types. These multifaceted actions have the potential to enhance the resilience of nerve cells, reduce inflammation, and enhance vascular health, collectively offering the prospect of slowing the clinical progression of Alzheimer’s disease (NCT04777396) [150].

##### Preventing Neuronal Cell Death and Neurotoxic Damage

Neuronal apoptosis, induced by β-amyloid and stress, is considered a significant pathophysiological marker of Alzheimer’s disease (AD) [151]. The results of postmortem brain research of patients with Alzheimer’s disease reveal a significant reduction of the volume of the cerebral cortex, a reduction of the size of the gyri by up to half, and an increase in the volume of the furrows [151].

Furthermore, prolonged exposure of neurons to Aβ led to the activation of caspase-3, which is a sign of apoptosis, as demonstrated by the detection of the active, cleaved form of caspase-3 through immunofluorescent staining. In neurons treated with Aβ oligomers, Western blot analysis showed an increase in the active form of caspase-3 and caspase-6, which play a significant role in the process of neuronal cell apoptosis [152]. Velmurugan et al. in their study demonstrated that activation of caspase-3 and caspase-6 by Aβ oligomers is significantly reduced by exendin-4 [152]. Perry et al. made a noteworthy discovery, indicating that both GLP-1 and exendin-4 can provide complete protection to cultured rat hippocampal neurons when they are subjected to glutamate-induced apoptosis [153]. Furthermore, exendin-4 was found to shield PC12 cells from apoptosis induced by β-amyloid (Aβ) [154]. Meanwhile, During et al. reported that [Ser (2) exendin (1–9)], an agonist of GLP-1 receptors, significantly reduced kainic-acid-induced apoptosis in the CA3 region of the hippocampus [155]. Yang et al. discovered a novel biological function of GLP-1, suggesting that the downstream signaling of stimulated GLP-1R can improve DNA repair capabilities by upregulating PI3K-AKT-CREB-mediated APE1 expression. This can alleviate DNA damage and cell death induced by ischemia [156]. Shi et al. showed that a novel GLP-1/GIP receptor dual agonist (DA-JC4) ameliorates cognitive decline by resensitizing insulin signaling, altering the inflammatory response, and reducing the ratio of pro-apoptotic BAX to anti-apoptotic Bcl-2 levels in streptozotocin (STZ)-induced AD rats [157].

Additionally, liraglutide has been reported to reduce cytotoxicity and apoptosis in SH-SY5Y cells under stress conditions induced by methylglyoxal, thapsigargin, and Aβ [158,159,160]. Similarly, in an Alzheimer’s disease (AD) model where SH-SY5Y cells were exposed to Aβ, semaglutide inhibited apoptosis by downregulating the expression of Bax induced by Aβ and upregulating the expression of Bcl2 suppressed by Aβ [161].

Animal studies have provided compelling evidence that the activation of GLP-1 receptors (GLP-1R) offers protection against endoplasmic reticulum (ER) stress and apoptosis triggered by amyloid. In the APP/PS1 (amyloid precursor protein/presenilin 1) animal model, the administration of a dual agonist targeting GLP-1R and GIPR through injection effectively restored cerebral Akt activity and Ser9-phospho-GSK-3β levels. These levels, which were previously reduced, were brought back to a state akin to those observed in control mice [108]. Although GLP-1 agonists and DPP4 and SGLT2 inhibitors are in widespread use in T2DM subjects, there is little clinical data presented here on the beneficial effects of these drugs on cognition or adverse events in either T2DM or MCI/AD subjects. The results of the phase II clinical trial (ELAD study, NCT01843075) involving over 200 patients with mild cognitive impairment (MCI) and Alzheimer’s disease who received liraglutide for one year are noteworthy. This study sheds light on the potential therapeutic benefits of liraglutide in the context of cognitive function and brain volume changes [162]. The results revealed that patients receiving liraglutide experienced improved glucose metabolism in the temporal lobes and an increase in cortical volume by MRI. Additionally, liraglutide improved cognitive functions as determined by ADAS-EXEC neuropsychological tests. The ability to protect cognitive function and preserve brain volume suggests that GLP-1 analogs like liraglutide may offer hope for improving the quality of life and delaying the progression of AD-related cognitive decline. Additionally, Nørgaard et al. (2022) have offered compelling evidence indicating that GLP-1 treatment in patients with type 2 diabetes could potentially open a new avenue for reducing the occurrence of dementia [163]. These findings are in line with observations made by Akimoto et al. (2020) [164].

##### Mitigating Oxidative Stress and Mitochondrial Dysfunction

In the context of Alzheimer’s disease, oxidative stress has emerged as the next contributor to the development and progression of this debilitating condition [165]. A multitude of research has illuminated the promising role of GLP-1 analogs in combating oxidative stress and mitigating the resultant cellular damage [108,134,166,167]. Research conducted on diabetic mice treated with GLP-1 analogs has yielded noteworthy results. These studies have shown significant reductions in biochemical markers associated with endoplasmic reticulum (ER) stress, alongside increased expression of antioxidant genes. Additionally, the treatment with GLP-1 analogs has led to improvements in metabolic parameters [168,169]. In in vitro studies, GLP-1 dual agonists, encompassing liraglutide, exendin-4, and GLP-1R/GIPR activators, have demonstrated their efficacy in safeguarding diverse cell types, including RGC-5 retinal cells, PC12 neurons, and SH-SY5Y cells, from the detrimental consequences of oxidative damage induced by hydrogen peroxide (H_2_O_2_). These compelling findings have received resounding validation through multiple research endeavors led by esteemed scholars such as Reich and Hölscher in 2022, Li et al., 2012, and Sharma et al., 2014, collectively underscoring the robust and consistent nature of these protective effects [108,134,135]. Furthermore, Chen et al. provided further substantiation of these findings by demonstrating the compelling ability of GLP-1 and exendin-4 to effectively alleviate change induced by oxidative stress within PC12 cells [166]. Spielman and colleagues showed that incretins reduced the level of oxidative stress in murine microglial cells (BV-2) by reducing ROS and NO and increasing the expression of antioxidant enzymes such as GPx1 and SOD1 [170].

Liraglutide, a GLP-1 analog, showcased its neuroprotective effects against AD-like neurodegeneration induced by hydrogen peroxide (H_2_O_2_) in the SH-SY5Y human neuroblastoma cell line [171]. Additionally, liraglutide has shown remarkable protective capabilities against brain β-amyloid accumulation in mouse models with early Alzheimer’s disease-like pathology. This protective effect extends to limiting oxidative stress and mitigating neuroinflammation, suggesting its potential as a therapeutic intervention for Alzheimer’s disease and related neurodegenerative conditions [172]. Furthermore, the treatment with exenatide has been shown to improve mitochondrial morphology, alleviate oxidative damage, correct mitochondrial energy deficits, and normalize mitochondrial dynamics. These compelling findings strongly indicate that exenatide, a medication already employed in clinical practice, holds significant promise as a therapeutic agent for Alzheimer’s disease by providing protection to crucial mitochondrial functions [167]. A study by Xie et al. revealed a new mechanism of GLP-1 neuroprotective action in Alzheimer’s disease [173]. In this study, liraglutide administered to 5×FAD mice for 8 weeks attenuated mitochondrial dysfunction and prevented neuronal loss by activating cyclic adenosine-3′,5′-monophosphate (cAMP)/phosphorylated protein kinase A (PKA) in the brain.

##### Mitigating Aβ Aggregation and Tau Protein Hyperphosphorylation

Emerging research on GLP-1 analogs has garnered significant interest due to their potential impact on crucial pathological processes in neurodegenerative diseases, particularly in addressing Aβ deposition and tau hyperphosphorylation. These advancements hold promise for enhancing cognitive and memory functions in individuals with neurodegenerative conditions. Perry et al. (2003) demonstrated the effectiveness of GLP-1 in reducing endogenous Aβ levels in the brain and decreasing amyloid precursor protein (APP) levels in neuronal cells. This suggests that GLP-1 analogs could potentially mitigate Aβ-related pathology in neurodegenerative diseases [174].

In a study by Xu et al., significant changes in hippocampal phosphorylated protein levels were observed in type 2 diabetic (T2D) rats after the use of exendin-4. Moreover, improvements were seen in the insulin signaling pathway in the brain. This was due to decreased PI3K/AKT activity and increased GSK-3β activity in the hippocampus of drug-intervention rats [175]. In their research, McClean and Hölscher discovered that liraglutide treatment resulted in a reduction in the levels of soluble Aβ oligomers. This potentially played an important role in the observed enhancements in memory in mice carrying the APP/PS1 mutations. Remarkably, liraglutide not only boosted memory but also reversed synapse loss and improved histological markers in mice with advanced neurodegenerative progression. These findings suggest that liraglutide has the capacity to alleviate existing impairments, even in mice at an advanced stage of degeneration [176]. Li et al. demonstrated that GLP-1 treatment leads to notable cognitive improvements in terms of learning and memory, which correlate with a reduction in both total and hyperphosphorylated tau levels [134]. In a preliminary study conducted on Alzheimer’s disease (AD) patients, treatment with exenatide over an 18-month period did not yield significant results in terms of cognitive measures, cortical thickness, volume, or biomarkers in biological fluids. However, there was a slight decrease observed in Aβ42 levels within neuronal extracellular vesicles (NCT01255163) [136]. In addition, research results suggest that drugs such as lixisenatide and the GLP-1 analog CJC-1131 have the potential to protect against Aβ suppression in the LTP range, which may indicate the ability of these compounds to maintain or restore normal synaptic function, which is crucial in the context of improving cognitive function and delaying AD progression [177,178].

Dulaglutide, a novel long-acting GLP-1 receptor agonist, has been found to ameliorate AD-like impairments in learning and memory. This effect is achieved by decreasing the hyperphosphorylation of tau and neurofilament (NF) proteins [179]. Studies show that liraglutide leads to reduced levels of total brain APP and Aβ oligomers in AD mice [176,180,181]. Furthermore, intervention with liraglutide can prevent tau hyperphosphorylation [182,183]. Song et al. confirmed in their research that exendin-4 has the ability to reduce the content of Aβ (1–42) both at the protein and mRNA level. In addition, a reduction in Aβ aggregation clusters (1–42) was observed in the Caenorhabditis elegans model of Alzheimer’s disease [184]. The accumulation of amyloid in AD is known to adversely affect synapses through various mechanisms. However, GLP-1 receptor activation has consistently demonstrated the ability to protect and potentially even restore synaptic function [108,165]. This suggests that GLP-1 analogs or interventions targeting the GLP-1 receptor pathway could have therapeutic potential in mitigating synaptic loss in AD. Mice lacking GLP-1R exhibit a learning impairment phenotype, which can be reversed by introducing the GLP-1R gene into their hippocampus, indicating the importance of this receptor in cognitive processes. Additionally, rats with increased expression of GLP-1R in the hippocampus demonstrate enhanced learning and memory capabilities, further highlighting the potential cognitive benefits associated with GLP-1 receptor activation [155].

These studies collectively suggest that GLP-1 analogs hold promise in addressing critical pathological processes in neurodegenerative diseases, offering potential benefits for cognitive and memory function enhancement.

### 3.2. Dipeptidyl Peptidase-4 (DPP-4) Inhibitors

#### 3.2.1. Mechanism of Action of Dipeptidyl Peptidase-4 (DPP-4) Inhibitors—Pharmacokinetic and Pharmacological Characterization

Dipeptidyl peptidase-4 (DPP-4), also known as CD26, made its initial appearance in scientific literature in 1966 as an enzyme endowed with the capacity to cleave dipeptides from the N-terminus of substrates containing proline and, to some extent, alanine at the penultimate position. This is particularly relevant in substrates such as GLP-1 and GIP [185,186]. Structurally, DPP-4 takes the form of a type II transmembrane serine protease, comprising two identical 110 kDa subunits interconnected through non-covalent interactions [187,188]. The human DPP-4, consisting of 766 amino acids, falls within the α,β-hydrolases family (Family S9B) [186]. Notably, DPP-4 finds its presence in a multitude of tissues and cell types, encompassing T-cells, B-cells, natural killer cells, epithelial cells, and endothelial cells. Furthermore, it has been detected in various organs, including the placenta, kidney, intestines, prostate, gallbladder, pancreas, and liver [186,188]. The active site of this enzyme resides within a capacious inner cavity, a product of the combined α/β hydrolase and β propeller domains [188].

The concept of inhibiting the DPP-4 enzyme as a strategy for treating type 2 diabetes mellitus (T2DM) was introduced around 1998 [186]. Presently, the armamentarium includes DPP-4 inhibitors such as sitagliptin, saxagliptin, vildagliptin, alogliptin, linagliptin, and gemigliptin. Dipeptidyl peptidase-4 (DPP-4) inhibitors, commonly referred to as gliptins, constitute a class of medications primarily employed for the management of type 2 diabetes mellitus (T2DM). They operate by targeting the DPP-4 enzyme, which plays a crucial role in glucose homeostasis and insulin regulation. DPP-4 is an enzyme naturally present in the body, particularly in various tissues and organs. Its primary function is to break down incretin hormones, such as glucagon-like peptide-1 (GLP-1) and glucose-dependent insulinotropic peptide (GIP). These incretins hold a vital responsibility in orchestrating blood sugar level regulation. They accomplish this by orchestrating the release of insulin in response to escalating glucose levels. However, the transient nature of these incretins is their Achilles’ heel, as DPP-4 swiftly dismantles them, curbing their effectiveness. These medications act as guardians against DPP-4’s enzymatic prowess. At therapeutic doses, all DPP-4 inhibitors can reduce plasma DPP-4 activity by 70-90%, resulting in an elevation of GLP-1 levels [189]. Notably, vildagliptin necessitates twice-daily administration to sustain its 24 *h* effect due to the requirement for a minimum of 80% DPP-4 activity inhibition to effectively increase GLP-1 and GIP levels, thereby eliciting a clinically relevant response [189] (Table 2).

This extension translates into a more potent release of insulin from pancreatic beta cells whenever blood sugar levels surge. Simultaneously, DPP-4 inhibitors rein in the secretion of glucagon, a hormone notorious for elevating blood sugar levels, directly from the pancreas. The net result is a finely orchestrated, glucose-dependent surge in insulin. This means insulin is released in response to high blood sugar levels, especially after meals, thereby curtailing post-meal spikes in blood sugar. Crucially, this mechanism minimizes the risk of hypoglycemia, a condition characterized by dangerously low blood sugar levels both in healthy people and in patients with T2DM [189]. Most importantly, GLP-1 analogs do not carry the risk of neuroglycopenia in patients with Alzheimer’s disease, which could potentially lead to cognitive decline in this group of patients [193].

Furthermore, DPP-4 inhibitors don an additional hat, potentially offering benefits that transcend glycemic control. Some of them have been linked to delaying gastric emptying, a phenomenon that decelerates the entry of glucose into the bloodstream following a meal. This added effect further bolsters post-meal blood sugar management [188].

Understanding the mechanism of action of DPP-4 inhibitors provides insights into the potential benefits of DPP-4 inhibitors beyond glycemic control, such as cardiovascular health and neuroprotection.

#### 3.2.2. Molecular Mechanisms Underlying the Neuroprotective Effects of DPP-4 Inhibitors

Amyloid-β peptide, hyperphosphorylation peptide, hyperphosphorylation of tau, and insulin abnormality are common pathological mediators and processes shared between Alzheimer’s disease (AD) and diabetes [108,194]. Some research reports that DPP-4 inhibitors may have a significant role in neurodegenerative and cognitive disorders.

##### Improvement of Insulin Sensitivity

Insulin resistance plays a key role in the pathogenesis of type 2 diabetes (T2DM) and may be associated with memory and learning disorders [12]. Brain insulin sensitivity leads to neurodegeneration due to the build-up of amyloid plaques. A number of studies show that DPP-4 inhibitors reduce insulin resistance of cells by inhibiting the degradation of the active amide GLP-1(7–36) [188,189]. As previously mentioned, GLP-1 performs a number of functions in the human body and, among others, is responsible for reducing the release of glucagon and increasing insulin sensitivity [98]. Therefore, the use of a DPP-4 inhibitor leads to an increase in the level of GLP-1, which, as confirmed by other studies on rodents with AD, has a positive effect on learning and improvement of cognitive functions [98]. In this respect, D’Amico et al., 2010 studied sitagliptin and showed that it increased GLP-1 levels in the brain, thereby improving cognitive function in mice [194]. Furthermore, research conducted by Siddiqui N. offers compelling evidence that treatment with linagliptin effectively mitigates brain insulin resistance and associated complications in the hippocampus [195,196]. Notably, the administration of linagliptin has also shown a positive impact on memory and motor responses. Beyond its cognitive enhancements and insulin resistance reduction, this antidiabetic medication has demonstrated the capacity to reduce the levels of Aβ senile plaques in the hippocampus, a pivotal pathological hallmark of Alzheimer’s disease. These findings collectively position linagliptin as a promising pharmacological therapy that holds potential for the prevention of Alzheimer’s disease symptoms [196].

##### Anti-Inflammatory Effects

Inflammatory processes in the central nervous system (CNS), such as increased levels of pro-inflammatory cytokines such as interleukin-1(IL-1), interleukin-6 (IL-6), and tumor necrosis factor (TNF-α), lead to neuronal cell death. IL-1, IL-6, and TNF-α are key substances secreted by activated microglia; their increased level in the body is characteristic of AD. In a study conducted by Kosaraju et al., it was observed that elevated levels of TNF-α and IL-1β were effectively reduced following treatment with DPP-4 inhibitors, such as vildagliptin. Additionally, in the context of this research, linagliptin was tested, and it demonstrated a dose-dependent capacity to significantly reduce the levels of neurotoxic cytokines within the hippocampus [196]. Moreover, in vivo studies involving mice revealed that the administration of sitagliptin at a dosage of 20 mg/kg resulted in a significant improvement in memory impairment. Sitagliptin, as a DPP-4 inhibitor, prolongs the activity of natural GLP-1 and enhances Aβ degradation, potentially exerting a beneficial effect in alleviating cognitive impairment associated with Alzheimer’s disease [194]. Importantly, this study provided confirmation that DPP-4 inhibition leads to a reduction in inflammation and nitrative stress within the brain, underscoring the neuroprotective effects associated with DPP-4 inhibitors [194].

##### Preventing Neuronal Cell Death and Neurotoxic Damage

Programmed cell death (PCD) is a process in which unnecessary and damaged cells can be eliminated, which allows for maintaining the physiological homeostasis of multicellular organisms. Incorrect activation of this process can cause cell death through various mechanisms, which induces pathological changes in neurons that can be observed in the pathogenesis of various neurodegenerative diseases, including Alzheimer’s disease [151]. DPP-4 inhibitors have a preventive effect on nerve cells, protecting them against degenerative changes [197,198,199,200]. Elbaz E. M. et al. showed that linagliptin treatment led to a significant decrease in behavioral and motor abnormalities in treated animals, indicating the amelioration of cuprizone-induced demyelination [197]. Neurotrophins, such as BDNF and NGF, play a crucial role in regulating the plasticity and survival of nerve cells. In a study by Zhang DD et al., it was discovered that in a diabetes model, levels of NGF and BDNF were decreased. However, treatment with vildagliptin resulted in an increase in these neurotrophins, effectively reversing the decline [199]. Furthermore, vildagliptin treatment has been found to significantly enhance memory and learning abilities in a rat model of diabetes induced by streptozotocin (STZ). Vildagliptin has been shown to boost synaptic plasticity by upregulating associated proteins, while simultaneously downregulating apoptosis and proteins linked to Alzheimer’s disease (AD) [198]. Additionally, the activation of Akt (Protein Kinase B) and the inhibition of GSK-3β (Glycogen Synthase Kinase-3β) likely played a role in improving cognitive function through vildagliptin treatment. These findings provide valuable evidence supporting the potential of vildagliptin as a therapeutic option for Alzheimer’s disease [198].

##### Mitigating Oxidative Stress and Mitochondrial Dysfunction

Oxidative stress in the brain plays an important role in the pathogenesis of AD. High levels of oxidative stress cause mitochondrial damage and an increase in reactive oxygen species (RO) results in cognitive decline [31,35,201]. Several in vivo studies have provided confirmation of the capability of sitagliptin and vildagliptin to effectively alleviate oxidative stress [198,199,200,201,202]. The findings from Pintana’s study demonstrated that both vildagliptin and sitagliptin have the capacity to restore mitochondrial function in the brain and enhance learning and memory in rats with induced insulin resistance resulting from a high-fat diet (HFD). The restoration of mitochondrial function in the brain after DPP-4 inhibitor treatment may be attributed to the extended presence of GLP-1’s activity [202]. Additionally, this study also showed that brain oxidative stress levels significantly decreased after treatment with vildagliptin and sitagliptin, which correlated with improved cognitive function and memory [202]. Furthermore, in preclinical trials, vildagliptin exhibited a significant impact on insulin sensitivity and mitochondrial function and enhanced hippocampal synaptic plasticity and cognitive abilities in an animal model of obesity [198].

These intriguing findings suggest that DPP-4 inhibitors, typically used as antidiabetic medications, may hold promise as a therapeutic agent for Alzheimer’s disease. They achieve this by safeguarding mitochondrial function and lowering the levels of detrimental reactive oxygen species in the brain.

##### Mitigating Aβ Aggregation and Tau Protein Hyperphosphorylation

It is a known fact that the formation and aggregation of Aβ plaques in hippocampal tissues leads to progressive cognitive decline in neurodegenerative diseases [108]. Saxagliptin, a DPP-4 inhibitor, prolongs the action of GLP-1, resulting in the reversal of cognitive deficits associated with reduced amyloid load, tau protein phosphorylation, and brain inflammation, as demonstrated by Kosaraju J. et al. (2013) [200]. The administration of saxagliptin for a 60-day period to animals with memory impairment induced by streptozotocin (STZ) led to a complete reversal of behavioral deficits in the treated animals. Additionally, animals treated with saxagliptin showed significantly increased GLP-1 levels in the hippocampus and a reduction in Aβ42 levels compared to the control group. The DPP-4 inhibitor effectively decreased the expression of both total tau and p-tau, underscoring the neuroprotective potential of this drug [200]. In the study by D’Amico et al., 2010, it was shown that long-term treatment of sitagliptin increased endogenous GLP-1 levels while decreasing the levels of amyloid-β peptide (Aβ) and amyloid precursor protein (APP) in the brain animals [194]. This discovery supports the idea that GLP-1 can reduce Aβ levels in the brain in vivo and decrease APP levels in cultured neuronal cells, as proposed by Reich and Hölscher in 2022 [108]. Moreover, research on human cells conducted by Kornelius et al. revealed that linagliptin dose-dependently inhibits Aβ-induced neurotoxicity and tau hyperphosphorylation by enhancing GLP-1 signaling. This effect appears to be associated with the activation of AMP-activated protein kinase (AMPK) through insulin signaling and the induction of antioxidant pathways, such as SIRT1. Clinical trials involving DPP-4 inhibitors have demonstrated that the use of these antidiabetic drugs is associated with a reduced amyloid burden and favorable long-term cognitive outcomes in diabetic patients diagnosed with Alzheimer’s Disease Cognitive Impairment (ADCI) [203,204].

The presented research results indicate the unique mechanism of DPP-4 inhibitors in the context of AD development.

### 3.3. Sodium-Glucose Cotransporter 2 (SGLT-2) Inhibitors

#### 3.3.1. Mechanism of Action of Sodium–Glucose Cotransporter 2 (SGLT-2) Inhibitors—Pharmacokinetic and Pharmacological Characterization

Sodium–glucose co-transporter 2 (SGLT-2) inhibitors are another group of new-generation antidiabetic drugs that have proven to be effective in the treatment of type 2 diabetes. These inhibitors target two distinct proteins, SGLT1 and SGLT2, which play pivotal roles in epithelial glucose co-transport processes. While SGLT1 primarily facilitates the absorption of dietary glucose in the gut, SGLT2 is responsible for reabsorbing the majority of filtered glucose within the kidney’s tubular system, with SGLT1 accounting for the remaining glucose reabsorption [205]. Since the US Food and Drug Administration’s approval of the first SGLT2 inhibitor in 2013, these compounds have become a fundamental component in the management of type 2 diabetes mellitus. This group includes drugs such as canagliflozin (Invokana), dapagliflozin (Farxiga), empagliflozin (Jardiance), and ertugliflozin (Steglatro).

SGLT2 inhibitors represent a unique class of anti-diabetic agents with an insulin-independent mode of action [206,207]. This insulin-independent mechanism of action allows for the administration of SGLT2 inhibitors to patients with various levels of beta-cell dysfunction or insulin resistance, minimizing the risk of hypoglycemia. The use of SGLT-2 inhibitors is so safe that these drugs do not cause hypoglycemia in patients with normal blood sugar levels [206,207]. SGLT2 inhibitors work by inhibiting the reabsorption of glucose in the kidneys, leading to increased urinary glucose excretion and reduced blood glucose levels [208]. It is important to note that the pharmacokinetic and pharmacodynamic properties of SGLT2 inhibitors may vary between different drugs in this class, and may also be influenced by factors such as age, gender, and renal function [61].

Absorption of SGLT-2 inhibitors is rapid, with peak plasma concentrations achieved within 1–2 h of oral administration. The bioavailability of these drugs ranges from 30–80% depending on the specific compound. The absorption of SGLT-2 inhibitors is not affected by food [60,209]. The volume of distribution for most compounds is relatively small, ranging from 50–100 L. The extent of protein binding varies among the different SGLT-2 inhibitors, with canagliflozin and empagliflozin exhibiting high levels of plasma protein binding (>90%), while dapagliflozin has a lower binding affinity (~91%) [60,61,209]. The half-life of SGLT-2 inhibitors is generally short, ranging from 6–13 h, and the drugs are eliminated primarily via the renal route [60,61,209]. SGLT2 inhibitors are generally safe and well-tolerated in patients with type 2 diabetes. However, like all medications, they are associated with certain risks and potential adverse effects. One of the most common side effects is an increased risk of urinary tract infections, including inflammation of the urinary tract, and genitals, orthostatic hypotension, and the risk of dehydration [62,63]. This group of antidiabetic drugs shows also favorable effects on the cardiovascular system, and it is especially recommended for patients with type 2 diabetes and diagnosed cardiovascular disease [205]. Treatment with SGLT-2 inhibitors is associated with reduced overall and cardiovascular mortality. In May 2023, the FDA extended dapagliflozin’s use to encompass the treatment of heart failure across the entire spectrum of left ventricular ejection fraction (LVEF) [65]. Furthermore, in addition to their effectiveness in improving glycemic control, SGLT-2 inhibitors have been linked to reductions in weight and blood pressure in individuals with type 2 diabetes mellitus (T2DM), whether used as a monotherapy or in combination with other antidiabetic medications [206,207]. SGLT2 inhibitors exhibit a nephroprotective effect by increasing the delivery of sodium to the distal portions of the kidneys and inhibiting tubular-glomerular feedback, which in turn lowers intraglomerular pressure and reduces albuminuria. Moreover, the disruption of glucose and sodium absorption in the proximal tubules results in the excretion of sodium in the urine. This leads to a decrease in effective blood volume, a reduction in blood pressure, and affects body weight through the loss of water. Additionally, SGLT-2 inhibitors influence inflammatory processes, reduce renal hypoxia, and impact mitochondrial metabolism in renal tissue [68].

#### 3.3.2. Physiological and Pharmacokinetic Attributes of SGLT-2 Inhibitors in the Central Nervous System—Mechanisms and Benefits

SGLT-2 inhibitors, originally designed for the management of type 2 diabetes (T2D), have garnered significant attention in recent years due to their potential benefits beyond glycemic control. Emerging research has shed light on the presence of SGLT-2 receptors in various areas of the body, including the central nervous system. This has prompted investigations into the mechanisms and potential advantages of SGLT-2 inhibitors within the central nervous system (CNS) [11,12]. Recent studies have identified SGLT-2 receptors in choroid plexus epithelial cells and ependymal cells of the brain. These receptors play a role in glucose transport and brain homeostasis, indicating a potential influence on brain function [11]. Furthermore, research has shown that SGLT-2 inhibitors may have a role in the context of neurodegenerative conditions, such as Alzheimer’s disease (AD). Recent studies suggest that SGLT-2 inhibitors may have neuroprotective effects, including the reduction of amyloid beta accumulation in the brain, which is a hallmark of Alzheimer’s disease. Additionally, SGLT-2 inhibitors have been shown to improve cerebral blood flow and increase the production of brain-derived neurotrophic factor (BDNF), which is involved in the growth and survival of neurons. The minimal metabolism of SGLT-2 inhibitors, coupled with their renal elimination, also suggests a lower risk of drug interactions and potential toxicity, which is especially important in the elderly population often affected by Alzheimer’s disease [61].

Impaired glucose metabolism and insulin signaling are recognized factors in the development and progression of AD, often manifesting several years before the initial clinical symptoms evident in this disease [13]. Moreover, higher glucose concentrations in brain tissue are associated with a more intense process of accumulation of both amyloids and pathological neurofibrillary changes. SGLT-2 inhibitors have been found to enhance glucose metabolism, improve insulin sensitivity, and reduce the accumulation of beta-amyloid plaques and neurofibrillary tangles in animal models, suggesting a potential neuroprotective effect [14].

Some research suggests that SGLT-2 inhibitors may influence atherosclerotic risk factors and pathways involved in both the acute and late stages of stroke, indicating a broader impact on cerebrovascular health [12]. Notably, a 2020 study conducted by Hierro-Bujalance et al. delved into the impact of empagliflozin, a representative drug from the SGLT2 inhibitor group, on vascular damage and impairment in a mixed murine model of Alzheimer’s disease and type 2 diabetes. This research highlighted the promising therapeutic potential of SGLT2 inhibitors in this context [17]. In 2021, Pawlos et al. delved deeper into the mechanisms and specific attributes that may underlie the neuroprotective potential of SGLT-2 inhibitors. This research provided valuable insights into the intricate pathways through which these medications might offer benefits in the realm of neurodegenerative diseases [13]. The relevant pharmacokinetic parameters of SGLT-2 inhibitors are listed below in Table 3.

#### 3.3.3. Molecular Mechanisms Underlying the Neuroprotective Effects of SGLT-2 Inhibitors’ Improvement of Insulin Sensitivity

Exciting recent discoveries have unveiled the pivotal role of SGLT-2 inhibition in reshaping our understanding of insulin sensitivity in peripheral tissues, a breakthrough brought to light by the research efforts of Merovci et al., 2014 and Yaribeygi et al., 2020. What makes this revelation even more captivating is the intricate web of molecular mechanisms at play, underscoring the complexity of the processes involved [221,222].

SGLT-2 inhibitors (SGLT-2i) possess a remarkable capability to combat the perils of glucose toxicity by promoting the excretion of excess glucose through urine. This action serves a dual purpose: it lowers blood glucose levels and serves as a countermeasure against the insidious effects of glucotoxicity. Glucotoxicity is a consequence of chronic hyperglycemia, a common adversary in diabetes. This menacing presence has long been associated with the impairment of beta-cell function and its correlation with insulin resistance, making it a formidable opponent. What is particularly striking is the compelling evidence stemming from research by Merovci et al., 2014 and List et al., 2011, demonstrating the potent anti-glucotoxic effects of SGLT-2 inhibition, with a special spotlight on dapagliflozin [221,223]. This inhibition not only alleviates the burden of glucotoxicity but also paves the way for enhanced glycemic control and an uplift in insulin sensitivity. Perhaps the most intriguing aspect is that these anti-glucotoxic effects are not ephemeral; they persist even after the cessation of treatment, emphasizing their profound significance [221].

Caloric disposition, a well-documented effect of SGLT-2i therapy resulting from the increased urinary excretion of glucose [224], contributes to weight loss and a reduction in circulating lipids [224,225,226]. Research confirms that SGLT-2i therapy effectively reduces body weight in individuals with diabetes [226]. Reduced adipose tissue mass and lower levels of circulating lipids are closely linked to improved insulin sensitivity [227]. It is worth noting that obesity-induced inflammatory responses, mediated by adipocytes and adipokines, are pivotal in the development of insulin resistance [228]. In this context, SGLT-2i therapy emerges as a promising avenue for reversing the adverse effects of a high-fat diet and obesity on insulin resistance, as exemplified by studies conducted by Shamansurova [229] and Xu [230].

Compelling evidence suggests that the “visceral fat lowering” effect of SGLT-2i significantly enhances insulin sensitivity by alleviating lipotoxicity, with empagliflozin standing out as a potent agent in this regard. Empagliflozin orchestrates a symphony of effects, including the modulation of inflammatory processes, the reduction of visceral fat, and the promotion of browning in adipose tissue [231]. Multiple clinical trials, some involving canagliflozin, further underscore the idea that SGLT-2i therapy enhances insulin resistance through the dual action of weight loss and the reduction of visceral fat, ultimately culminating in improved metabolic health [232].

##### Anti-Inflammatory Effects

A pivotal aspect of Alzheimer’s disease (AD) is neuroinflammation, characterized by microglial activation and the release of pro-inflammatory cytokines that contribute to synaptic dysfunction and neurotoxicity [233,234]. Wang et al. (2015) have posited that pro-inflammatory cytokines released by microglia play a substantial role in the onset and progression of AD [39]. SGLT-2 inhibitors offer an intriguing avenue for intervention by inhibiting or reducing inflammatory processes through various molecular pathways. These pathways encompass direct effects on the immune system, modulation of the renin–angiotensin system (RAS), alterations in hemodynamic changes, modulation of immune system function, and adjustments to the redox state in tissues [230]. The anti-inflammatory properties of SGLT-2 inhibitors hold great promise for the development of innovative therapeutic approaches for neuroinflammatory conditions like AD.

According to a study by Lathe et al. (2014), atherosclerosis, a systemic inflammatory disease affecting blood vessels, and Alzheimer’s disease (AD) may share a common pathogenic basis [235]. Macrophages, through their involvement in inflammation and A-beta clearance, play a critical role in the pathogenesis of both atherosclerosis and AD [140]. Furthermore, AD’s pathophysiology has been associated with the NLRP3 inflammasome, a crucial component of the innate immune response. Upon activation, it leads to the release of pro-inflammatory cytokines such as IL-1 and IL-18, exacerbating neuroinflammation and promoting the deposition of A-beta plaques [236]. Inhibition of the NLRP3 inflammasome has shown promise in reducing cognitive impairment associated with AD, as suggested by Tejera et al. (2019) and Lonnemann et al. (2020) [236,237].

The potential for SGLT2 inhibitors to provide anti-inflammatory benefits has garnered attention. Studies have demonstrated that the SGLT2 inhibitor canagliflozin reduces biomarkers associated with fibrosis and inflammation [238]. In the mentioned study, canagliflozin treatment led to lower levels of TNFR1, IL-6, MMP7, and FN1 in clinical trial settings compared to glimepiride treatment. This suggests that canagliflozin therapy may help reverse inflammatory processes at the molecular level [239]. This information is reinforced by other investigations. In a 2021 experiment that explored the effects of empagliflozin and gemigliptin (a DPP-4 inhibitor used to treat diabetes) on macrophage inflammatory responses, it was discovered that empagliflozin exhibited anti-inflammatory properties. The underlying signaling mechanisms for these effects were also elucidated, as the IKK/NF-B, MKK7/JNK, and JAK2/STAT1 signaling pathways in macrophages were inhibited [240].

##### Preventing Neuronal Cell Death and Neurotoxic Damage

Sodium–glucose cotransporter 2 inhibitors (SGLT2i), primarily recognized for their antidiabetic effects, have recently emerged as potential protectors against neuronal cell death and neurotoxic damage [241]. In this context, exploring their multifaceted activities becomes crucial.

Cerebrovascular dysfunction is a brain condition linked to vascular pathology, primarily characterized by altered blood flow in the brain. In glucose metabolism disorders, the brain’s microvascular system is compromised, leading to neurovascular remodeling that disrupts neurons, astrocytes, pericytes, and endothelial integrity, thickens the basement membrane, and results in myelin loss. Furthermore, the development of neuronal damage has been associated with increased sodium influx, as demonstrated in SGLT-1 knockdown mice by Yamazaki et al. (2017) [242]. Additionally, Ishida et al. (2020) reported that sodium influx during ischemia is mediated by SGLT1 receptors, leading to depolarization and promoting neuronal cell death [243]. Yamazaki and colleagues (2017) found that deleting the SGLT1 receptor was linked to reduced production of proinflammatory cytokines and improved cognitive function in a mouse model of subcortical white matter infarction with cognitive impairment [242]. Sim et al. (2021) confirmed the significant role of SGLT receptors in cerebral ischemia-reperfusion injury [244]. Some studies have shown that SGLT-2 inhibitors may reduce cognitive decline and brain damage in stroke patients. Despite the impact of SGLT2 inhibitors on significant cerebrovascular risk factors, such as hyperglycemia, hypertension, obesity, dyslipidemia, and atherosclerosis, Usman et al. (2018) found that these inhibitors did not reduce the incidence of ischemic stroke [245]. Hierro-Bujalance and colleagues assessed the effect of empagliflozin on vascular damage in a mixed murine model of AD and T2DM in a study published in 2020. Their results indicated that EMP therapy improved cognitive impairments in APP/PS1xdb/db mice by reducing bleeding, microglial loads, and adverse neurovascular alterations, which shows that there is evidence for the ability of SGLT-2 inhibitors to modulate inflammation-relevant microglia in AD brains. [246]. An extremely interesting finding is that the use of SGLT-2 inhibitors in patients suffering from type 2 diabetes (T2DM) is associated with a lower risk of dementia compared to the group of patients taking DPP-4 inhibitors [247].

This data suggests a potential neuroprotective role for SGLT-2 inhibitors in addressing cerebrovascular-related cognitive impairments and neuronal damage in the context of Alzheimer’s disease.

##### mTOR Activation and Its Impact on Catabolic Pathways

Uncontrolled mTOR up-regulation has been linked to the complex molecular chain that underlies Alzheimer’s disease (AD). There is a significant correlation between mTOR activation and tau and amyloid protein hyperphosphorylation and aggregation, which are hallmarks of AD pathology [248]. Additionally, the “Endo-Lysosomal Dysfunction” concept of Alzheimer’s disease is supported by the disruption of lysosomal protein breakdown pathways caused by persistent mTOR activation [249]. This highlights the potential importance of mTOR regulation in addressing cognitive impairment in AD.

Recent studies showed that SGLT-2 inhibitors can affect the mTOR pathway and, as a result, function in reducing cognitive impairment linked to AD. By encouraging glucose loss through urine, glycogenolysis, and gluconeogenesis, SGLT-2 inhibitors are reported to produce a catabolic state, making the transition from anabolic to catabolic metabolism easier [249]. These outcomes mimic the effects of fasting and are accompanied by transcriptional adjustments that resemble fasting, including activation of SIRT/AMPK and inhibition of Akt/mTOR [250]. The circadian regularity of mTOR activation offers an intriguing option for intervention. It is suggested that restoring this rhythm may be helpful in treating metabolic disorders and cognitive loss. Increased physical activity, calorie restriction, and intermittent fasting have all been proposed as potential mTOR cycling regulators [248]. Studies have demonstrated that SGLT2 inhibitors possess the ability to suppress mTOR and restore its circadian rhythm [251]. This modulation of the mTOR pathway aligns with the proposed benefits of restoring circadian mTOR rhythm to counteract cognitive impairment [252]. SGLT2 inhibitors hold particular promise in light of their potential to address the challenges associated with implementing lifestyle interventions in clinical practice. By providing an alternative means of achieving catabolic states and regulating mTOR, these inhibitors offer a novel avenue for managing cognitive decline in metabolic diseases, aligning with hypotheses like the “Type 3 Diabetes Hypothesis”, “Mitochondrial Cascade Hypothesis”, and “Endo-Lysosomal Dysfunction Hypothesis” [248].

##### Mitigating AB Aggregation and Tau Protein Hyperphosphorylation

A pivotal pathological mechanism in Alzheimer’s disease (AD) involves the aggregation of beta-amyloid plaques and neurofibrillary tangles (NFTs) comprising tau protein, which binds to microtubules [253,254]. Recent investigations propose that mitigating these processes is possibly achievable through sodium–glucose co-transporter 2 (SGLT-2) inhibition. Hierro-Bujalance et al. [245] conducted a pioneering study on the SGLT2 inhibitor empagliflozin (EMP), demonstrating a reduction in senile plaque density and overall levels of soluble and insoluble amyloid β (Aβ) in the cortex and hippocampus of APP/PS1xd/db mice. EMP also exhibited positive effects on cognitive abilities, ameliorating memory impairment. Notably, EMP administration limited tau phosphorylation in the cortex, though statistical significance in the hippocampus remained elusive, contrasting results seen with incretin-based diabetes mellitus (DM) treatments [241]. Lin et al. (2014) reported EMP’s prevention of cognitive function impairment in db/db mice, attributing this effect to a decrease in cerebral oxidative stress and an increase in cerebral brain-derived neurotrophic factor (BDNF), known for its neuroprotective properties [21]. Furthermore, a prospective study by Mone et al. (2022) provided significant evidence for the positive impact of SGLT-2 (EMP) inhibitors on cognitive function in older patients with diabetes [255].

SGLT2 inhibitors may assist patients with Alzheimer’s disease due to their direct neuroprotective effects, such as BDNF increase and AChE inhibition, as well as anti-inflammatory, anti-oxidative, or atheroprotective effects [256]. In a scopolamine-induced memory impairment rat model, Arafa et al. observed the beneficial effects of another SGLT-2 inhibitor, canagliflozin, suggesting a potential acetylcholinesterase inhibitory activity as an additional property of SGLT2 inhibitors [257]. Molecular docking studies on dapagliflozin supported this notion, indicating dual inhibitor properties against acetylcholinesterase and SGLT-2 [257]. These findings align with the outcomes of previous studies on other SGLT inhibitors, highlighting a promising avenue for addressing AD pathology through SGLT-2 inhibition.

Additionally, by increasing brain insulin sensitivity, SGLT-2 inhibitors may benefit AD patients. In eight out of ten Alzheimer’s patients, insulin resistance is prevalent [258]. Since the glucose metabolic rate is decreased in the brains of AD patients during fluorodeoxyglucose positron emission tomography (FDG PET), peripheral resistance to insulin also develops in the central nervous system (CNS) [258,259]. Additionally, insulin resistance was linked to GSK3-signaling activation, which contributes to tau phosphorylation, Aβ generation, and Aβ-mediated neuronal injury. SGLT-2 inhibitor therapy significantly decreased tau phosphorylation and the density of senile plaques in AD pathogenesis in earlier research using mouse models. This effect was connected to an improvement in cognitive processes, such as memory and learning, in the Morris water maze test and the new object identification test.

## 4. Conclusions

The analysis of available research results regarding the effects of antidiabetic drugs on Alzheimer’s disease pathology provides a robust foundation for optimism. Antidiabetic drugs, initially designed to manage diabetes, have emerged as a ray of hope in the field of neurodegenerative research. What makes these medications truly remarkable is their capacity to modulate crucial molecular pathways and neurochemical processes intricately linked to cognitive function and the development of Alzheimer’s disease, such as the reduction of amyloid-*β* levels, tau hyperphosphorylation, and the amelioration of inflammation and oxidative stress. By addressing these multifaceted aspects, these antidiabetic drugs present a comprehensive approach towards potentially ameliorating cognitive decline and the neurodegenerative processes in individuals dealing with both diabetes and Alzheimer’s disease. The improvements in cognitive functions, particularly in learning and memory, attributed to GLP-1 receptor agonists open a promising avenue for intervention. Moreover, the demonstrated reduction in oxidative stress and chronic inflammation in the brain, driven by the use of GLP-1 analogs, underscores their potential as neuroprotective agents. Similarly, DPP-4 inhibitors, which extend the action of GLP-1, exhibit comprehensive effects in reducing amyloid-*β* occurrence, τ hyperphosphorylation, and inflammation. The observed enhancements in spatial learning, memory capacity, and overall cognition further solidify the case for DPP-4 inhibitors as potential cognitive enhancers. Additionally, the reduction in oxidative stress contributes an additional layer of neuroprotective benefit. SGLT-2 inhibitors, while showing a more limited impact, still make a significant contribution to the reduction of hyperphosphorylated tau levels, decreased amyloid-β occurrence, and improved cognitive function. The mitigation of oxidative damage and the reduction of inflammatory processes offer further support for the potential role of SGLT-2 inhibitors in the treatment of Alzheimer’s disease.

Our analysis highlights the significant potential benefits offered by each of these three classes of diabetes medications when used in individuals with Alzheimer’s disease. Nonetheless, it is crucial to acknowledge the intricate and diverse nature of Alzheimer’s disease cases. The process of selecting the most appropriate drug is a multifaceted decision influenced by a multitude of factors. This process involves delving into the intricacies of an individual patient’s unique medical profile, considering their existing comorbidities, and gaining a deep understanding of their individual response to medication. This comprehensive approach underscores the paramount importance of conducting a thorough evaluation of each patient’s specific circumstances when making this pivotal decision. This realization emphasizes the critical need for ongoing research and the implementation of a personalized treatment approach. Such an approach carries profound significance, especially within the broader context of advancing innovative strategies for managing the complex and multifaceted nature of Alzheimer’s disease. Discovering novel methods of intervention has the potential to greatly enhance the quality of life for patients and their families who are grappling with this challenging situation. 

## Figures and Tables

**Table 1:** Pharmacokinetic characteristics of glucagon-like peptide-1 (GLP-1) receptor agonists.

Parameters	Glucagon-like Peptide-1 (GLP-1) Receptor Agonists				
	Liraglutide	Exenatide	Dulaglutide	Semaglutide	Lixisenatide
Molecular Weight [kDa]	3.8	3.7	59.7	4.1	3.3
Cmax	1.6 ng/mL	300 ug/mL	51.6 ng/mL	3.4 ng/mL	35 ng/mL
The volume of distribution [L]	11–17	28	3.09–6	8–9.4	100
Tmax [h]	9–12	2.4	90	165–184	3
Bioavailability [%]	55	22–25	47–65	89	N/A
Ability to cross the BBB	YES	YES	NO	NO	YES
References	[106,107,108]	[106,109,110,111,112,113,114]	[106,115]	[106,116,117,118]	[106,110,119,120,121]

Explanation of abbreviations: Cmax, maximum plasma concentration; Tmax, time to reach maximum concentration; NA, not available.

**Table 2:** Pharmacodynamic and pharmacokinetic properties of DPP-4 inhibitors in clinical use.

Parameters	DPP-4 Inhibitors					
	Sitagliptin	Vildagliptin	Saxagliptin	Alogliptin	Linagliptin	Gemigliptin
Molecular Weight [g/mol]	407.31	303.4	315.417	339.399	472.553	489.370
Cmax	491.7 (135.9) ng/mL	308 ± 67 ng/mL	640 (21) ng/mL	57.8 ng/mL	11–12 nmol/L	62.7 ng/mL
The volume of distribution [L]	198	71	151	417 L	1110 L	fill
Tmax [h]	2,5	1.75	2	1,5	2	1.8
Bioavailability [%]	87%	85%	75%	100%	30%	63%
Ability to cross the BBB	YES	YES	YES	YES	YES	N/A
Half-life	12.4 h		2.5 h, 3.1 h (active metabolite)	21 h	12 h	17.1
The primary route of elimination	Urine		Bile duct, urine	Urine, feces	Feces, urine	Urine, feces
References	[189,190,191,192]					

Explanation of abbreviations: Cmax, maximum plasma concentration; Tmax, time to reach maximum concentration; NA, not available.

**Table 3:** Pharmacokinetic characteristics of sodium–glucose cotransporter 2 inhibitors.

Parameters	SGLT-2 Inhibitors					
	Dapagliphlozin	Canagliphlozin	Empagliphlozin	Ipragliphlozin	Luseogliphlozin	Tofogliphlozin
Molecular Weight [kDa]	408.9	444.5	450.9	404.5	434.5	386.4
Cmax	2465 nmol/L for the 100 mg dose 7828 nmol/L for the 300 mg dose	2465 nmol/L for the 100 mg dose 7828 nmol/L for the 300 mg dose	259 nmol/L for the 10 mg dosxe 687 nmol/L for the 25 mg dose	1277 ± 360a	NA	NA
The volume of distribution [L]	118	119	74	NA	39	50
Tmax [h]	1.5	1.5	1	1.5	1.5	0.75
Bioavailability [%]	78%	65%	86%	90.2%	NA	97.5
Ability to cross the BBB	YES	YES	YES	YES	YES	YES
Half-life	12.9 h	10.6 h for the 100 mg dose 13.1 h for the 300 mg dose	12.4 h	14.97 h	NA	NA
The primary route of elimination	feces/urine					
References	[210,211,212,213,214]	[120,210,214]	[210,211,214]	[210,214,215,216]	[210,214,217]	[210,214,218,219,220]

Explanation of abbreviations: Cmax, maximum plasma concentration; Tmax, time to reach maximum concentration; NA, not available.

## Data Availability

Not applicable.
